# Informing the Alfred Registry for Emergency Care Project: An analysis of presenting complaint documentation in an emergency department

**DOI:** 10.1111/1742-6723.13984

**Published:** 2022-04-20

**Authors:** Matthew White, Gerard M O'Reilly, Rob D Mitchell, Michael Noonan, Ryan Hiller, Biswadev Mitra, Andrew Paton, Kathryn Pristupa, Carl Luckhoff, De Villiers Smit, Peter A Cameron

**Affiliations:** ^1^ Central Clinical School Monash University Melbourne Victoria Australia; ^2^ Austin Health Melbourne Victoria Australia; ^3^ Emergency and Trauma Centre Alfred Health Melbourne Victoria Australia; ^4^ School of Public Health and Preventive Medicine Monash University Melbourne Victoria Australia; ^5^ National Trauma Research Institute Alfred Health Melbourne Victoria Australia; ^6^ Trauma Service, Alfred Health Melbourne Victoria Australia; ^7^ Adult Retrieval Victoria, Ambulance Victoria Melbourne Victoria Australia; ^8^ Nursing and Midwifery Monash University Melbourne Victoria Australia

**Keywords:** emergency, registry, presenting complaint, quality, health information

## Abstract

**Objective:**

To assess the feasibility of an ED presenting complaint (PC) tool that categorised all ED PCs into 10 categories.

**Methods:**

A retrospective analysis of 1445 consecutive patient encounters was conducted. The primary outcome was the frequency of use of the 10 PC categories.

**Results:**

Of the 1203 patient encounters meeting inclusion criteria, the PC tool was completed by clinicians in 574 (47.7%). When completed, the tool's 10 options were selected for most presentations (72.3%).

**Conclusion:**

The PC tool captured the majority of presenting complaints in 10 categories. External validation is recommended.

## Introduction

Patient registries are a useful tool for improving hospital systems and synthesising data on patient outcomes. Clinical quality registries provide an opportunity for continual improvement of patient outcomes but are rare in emergency care. The ED presents unique challenges in the implementation of patient registries because of the variety of presenting complaints (PCs), urgency of care and patient comorbidities. Despite the challenges associated with ED clinical quality registries, there remains clear value in documenting and categorising PCs to monitor the processes of care and outcomes for ED patients.^1^

In response to the COVID‐19 pandemic, the Alfred Registry for Emergency Care (Alf‐REC) was established to evaluate the outcomes of patients presenting to the Alfred Health Emergency and Trauma Centre, improve hospital systems and disease surveillance.^2^

In the absence of an existing validated, consensus‐based approach to classifying ED PCs, the Alf‐REC Project developed a bespoke PC tool.^2^ Following expert consultation and consensus, 10 different PC categories were identified using the Hanlon Method for Prioritising Health Problems.^3^ Factors considered were presentation frequency, need for emergency intervention and/or being a signal for an infection of epidemic potential. ED practitioners completing an electronic medical record (EMR) entry were asked to also complete this tool. The tool was nested as a drop‐down box at the top of the Alfred Health's dedicated emergency presentation note. Tool completion was not mandatory.

Approximately 9 months after PC tool implementation, an evaluation was conducted. The primary aim of the present study was to determine the proportion of presentations which were captured by the 10 selected PC categories.

## Methods

The present study was completed at The Alfred Emergency and Trauma Centre, an adult tertiary hospital ED. All patient health records for the 7‐day period from 08.01 hours on 12 March 2021 to 08.00 hours on 19 March 2021 were accessed via the hospital EMR database. All patients with a completed emergency presentation note were included. There were 10 PCs available for selection, with an 11th code “Other” used as an alternative. For each patient, the selected PC category was recorded. The primary outcome was the proportion of cases for which each PC was selected; the sampling of a complete week was based on generating simple descriptive statistics for this primary outcome.

Tool completeness was reported by frequency and the association between tool completeness and triage category, time of presentation and physician role, respectively.

The Alfred Health ethics approval number for the Alf‐REC Project is 282/20.

## Results

There were 1445 patient presentations to ED during the present study period; 1203 (83.3%) had a dedicated emergency presentation note completed. The remaining 242 (16.7%) patients included those who did not wait to be seen or for whom the clinician used an alternative EMR note which did not include the PC tool. The non‐compulsory PC tool was completed for 574 (47.7%) encounters.

Table 1 summarises PC tool completion, patient arrival time, triage category and physician. There was an association between PC tool completion and both patient arrival time and physician role; there was no association with triage category.

**TABLE 1:** Patient arrival time, triage category and physician role completing the presenting complaint (PC) tool

Variable	PC box ticked, *n* = 574	PC box not ticked, *n* = 629	Emergency presentation note not used, *n* = 242	Odds ratio (95% CI)†
Arrival time, h				
00.01–08.00 (Reference group)	72 (33.0)	109 (50.0)	37 (17.0)	–
08.01–16.00	295 (45.2)	279 (42.7)	79 (12.1)	1.6 (1.1–2.2)
16.01–24.00	207 (36.1)	241 (42.0)	126 (22.0)	1.3 (0.9–1.8)
Triage category				
Triage 1	7 (50.0)	6 (42.9)	1 (7.1)	0.9 (0.3–3.2)
Triage 2	109 (46.2)	112 (47.5)	15 (6.4)	0.8 (0.4–1.5)
Triage 3	283 (42.2)	300 (44.7)	87 (13.0)	0.7 (0.6–1.1)
Triage 4	153 (33.9)	194 (43.0)	104 (15.5)	0.6 (0.3–1.2)
Triage 5 (Reference group)	22 (29.7)	17 (23.0)	35 (47.3)	–
Physician role				
Consultant (Reference group)	64 (33.7)	126 (66.3)	–	–
Fellow/Registrar	263 (47.0)	296 (53.0)	–	1.7 (1.2–2.4)
HMO	90 (47.6)	99 (52.4)	–	1.8 (1.2–2.7)
Intern	80 (65.0)	43 (35.0)	–	3.7 (2.3–5.9)
Student	15 (78.9)	4 (21.1)	–	–
Nurse Practitioner	51 (46.8)	58 (53.2)	–	1.7 (1.1–2.8)
Physiotherapist	11 (78.6)	3 (21.4)	–	–

† Odds ratio not reported for small numbers because of imprecision.

Patients who arrived between 08.01 hours and 16.00 hours had the PC tool completed more frequently than for other presentation times. More experienced clinicians completed the tool less frequently.

Table 2 lists the frequency (%) of selection for each PC category. “Acute injury” (28.4%) was the most frequently selected, with “Diarrhoea” (1.1%) the least common. There were 159 (27.7%) cases where the option “Other” was selected.

**TABLE 2:** Presenting complaint frequency

Presenting complaint	Frequency, *n* (%)
Acute injury	163 (28.4)
Abdominal pain	73 (12.7)
Chest pain	65 (11.3)
Mental health	34 (5.9)
Shortness of breath	27 (4.7)
Fever	22 (3.8)
Collapse	22 (3.8)
Decreased Glasgow Coma Scale	18 (3.1)
Limb weakness	10 (1.7)
Diarrhoea	6 (1.1)
Other	159 (27.7)

For 23 (1.5%) cases, more than one presenting complaint was selected.

## Discussion

This short report evaluated the introduction of a simple PC tool to determine the extent of PC capture. Completion of this added PC tool was not mandated and therefore overall completeness was moderate. Importantly, where the tool was utilised, its 10 categories captured the PC group of almost three quarters of patient presentations.

Regarding completeness for this non‐compulsory PC tool, the most junior medical staff (not rostered overnight) were the most compliant. Previous work has similarly demonstrated superior documentation completion among junior clinicians.^4^

Although the tool performed well in terms of PC capture, further analysis is required to determine whether the “Other” category includes a significant number of clinically important PCs to form one or more additional PC codes in the tool. Future research is planned to further validate, including externally, at different healthcare centres with different patient bases (including paediatric and women's health populations), and improve the PC tool, as PC‐related patient pathways, rather than retrospective diagnoses, will better monitor ED demand and inform emergency care system policy.

## Conclusion

The 10 categories used in this feasibility study facilitated categorisation of the majority of presentations to a tertiary hospital ED. These findings will inform further refinements to the PC tool.

## Data Availability

The data that support the findings of this study may be available on reasonable request from the corresponding author. The data are not publicly available due to privacy or ethical restrictions.
